# Post-Treatment Plasma D-Dimer Levels Are Associated With Short-Term Outcomes in Patients With Cancer-Associated Stroke

**DOI:** 10.3389/fneur.2022.868137

**Published:** 2022-04-04

**Authors:** Sho Nakajima, Hiroyuki Kawano, Kazuo Yamashiro, Ryota Tanaka, Tomoaki Kameda, Naohide Kurita, Kenichiro Hira, Nobukazu Miyamoto, Yuji Ueno, Masao Watanabe, Teruyuki Hirano, Shigeru Fujimoto, Takao Urabe, Nobutaka Hattori

**Affiliations:** ^1^Department of Neurology, Juntendo University School of Medicine, Tokyo, Japan; ^2^Department of Neurology, Juntendo University Urayasu Hospital, Chiba, Japan; ^3^Department of Stroke and Cerebrovascular Medicine, Kyorin University, Tokyo, Japan; ^4^Division of Neurology, Department of Internal Medicine, Jichi Medical University, Tochigi, Japan; ^5^Department of Neurology, Shin-Oyama City Hospital, Tochigi, Japan

**Keywords:** cancer, D-dimer, stroke, anticoagulant, prognosis

## Abstract

**Background and Objective:**

Hypercoagulability is associated with increased risks of ischemic stroke and subsequent mortality in patients with active cancer. This study investigated the relationships between plasma D-dimer levels after stroke treatment and short-term outcomes in patients with cancer-associated stroke.

**Methods:**

This retrospective, observational, multicenter study analyzed consecutive patients with cancer-associated ischemic stroke. Hypercoagulability was assessed by plasma D-dimer levels before and after stroke treatment. Short-term outcomes were assessed in terms of poor outcomes (a modified Rankin Scale score >3), cumulative rates of recurrent ischemic stroke, and mortality at 30 days after admission.

**Results:**

Of 282 patients, 135 (47.9%) showed poor outcomes. Recurrent ischemic stroke was observed in 28 patients (9.9%), and the cumulative mortality rate was 12.4%. Multivariate analysis showed that post-treatment plasma D-dimer levels ≥10 μg/ml were independently associated with both poor outcomes (adjusted odds ratio [OR], 9.61; 95% confidence interval [CI], 3.60–25.70; *P* < 0.001) and mortality (adjusted OR, 9.38; 95% CI, 3.32–26.44; *P* < 0.001). Pre-treatment plasma D-dimer levels ≥10 μg/ml were not associated with these outcomes. Patients who received heparin had higher pre-treatment plasma D-dimer levels than those treated with other anticoagulants. Heparin produced a significant reduction in D-dimer levels from pre- to post-treatment without increasing the incidence of hemorrhagic events.

**Conclusion:**

A high plasma D-dimer level after stroke treatment was associated with poor short-term outcomes in patients with cancer-associated stroke. Using anticoagulants to reduce D-dimer levels may improve short-term outcomes in these patients.

## Introduction

Patients with cancer have an increased risk of stroke (1–5). A recent systematic review indicated that the risk of arterial thromboembolism including myocardial infarction and ischemic stroke is highest immediately after the diagnosis of cancer and in patients with lung and pancreatic cancers (6). Furthermore, the risk of arterial thromboembolic events begins to increase before the diagnosis of cancer in older individuals (7). Patients with cancer have a high risk of either in-hospital mortality (8, 9) or long-term mortality (10) after stroke.

Cancer-associated stroke is attracting attention as an emerging subtype of ischemic stroke with unique pathomechanisms (11, 12). High plasma D-dimer levels are seen in patients with cancer-associated ischemic stroke, suggesting the importance of hypercoagulability as the etiology of the stroke (13–18). Accumulating data suggest that the plasma D-dimer level, a marker of activated coagulation and fibrinolysis, is a useful biomarker for the diagnosis of cancer-associated stroke and the evaluation of recurrent stroke risk (19, 20). Although previous studies have reported that anticoagulation therapies can effectively correct hypercoagulability in patients with cancer-associated stroke (21–23), data on the effects of anticoagulation therapy on prognosis remain limited. The aim of this study was to investigate the relationship between D-dimer levels after stroke treatment and short-term outcomes in patients with cancer-associated stroke.

## Methods

### Patients

This was a retrospective, observational, multicenter study of patients with cancer-associated stroke. Ethics approval was obtained from the Juntendo University Ethics Review Committee and the relevant ethics committees at all participating centers. All patients provided informed consent in compliance with institutional guidelines. We analyzed consecutive patients with acute ischemic stroke who were admitted to one of five stroke centers at University or city hospitals in Japan (Juntendo University Hospital, Juntendo University Urayasu Hospital, Kyorin University Hospital, Jichi Medical University Hospital, and Shin-Oyama City Hospital) between March 2011 and December 2019. The inclusion criteria were: (1) documented acute ischemic stroke based on diffusion-weighted imaging (DWI) within 48 h after symptom onset; (2) age 20 years or older; (3) known or newly diagnosed active cancer at the time of stroke diagnosis, after stroke, or during hospitalization; and (4) ischemic stroke that could not be explained by conventional mechanisms (large-artery atherosclerosis, cardioembolism, small-vessel occlusion) according to the Trial of ORG 10172 in Acute Stroke Treatment classification (24). Active cancer was defined as a diagnosis of or treatment for cancer during the 6 months preceding the stroke diagnosis, or the presence of recurrent or metastatic cancer (21). Patients with primary central nervous system tumors were excluded. Of the 7,029 patients with acute ischemic stroke admitted to our centers during the study period, 337 patients (4.8%) were diagnosed with cancer-associated stroke. Of these, 55 patients were excluded due to incomplete data, including lack of post-treatment plasma D-dimer levels. Only survivors whose D-dimer values were measured before and after stroke treatment were included in the analysis. A final total of 282 patients were analyzed in the present study.

### Clinical Assessments

We collected demographic and clinical data, including age, sex, cancer type and histology, presence of systemic metastasis, medical history, pre-admission treatment, National Institutes of Health Stroke Scale (NIHSS) score on admission, patterns of acute ischemic stroke on DWI, and stroke treatment, from medical records. According to stroke treatment, patients were classified into none, antiplatelet, warfarin, direct oral anticoagulant (DOAC), and heparin groups. Either continuous intravenous infusion of unfractionated heparin or twice-daily subcutaneous injection of heparin calcium was used in patients in the heparin group. Plasma D-dimer levels before and after stroke treatment were measured on admission and at a median of 19 days (interquartile range, 9–34 days) after admission, respectively. Short-term outcomes assessed were the modified Rankin scale (mRS) score, cumulative rates of recurrent ischemic stroke, and mortality 30 days after admission. Poor outcomes were defined as mRS score >3 (25). Recurrent ischemic stroke was defined as a new lesion detected by computed tomography or magnetic resonance imaging in patients with new acute neurological symptoms. Rates of hemorrhagic events and clinical outcomes were compared among the stroke treatment groups.

### Statistical Analysis

Continuous variables were compared using either Student's *t*-test or the Mann-Whitney *U*-test, as appropriate, after normality distribution testing. The frequency of categorical variables was compared using the χ^2^ test. This was a retrospective, observational study, and the treatment strategy was dependent on stroke neurologists' decisions based on the patients' conditions. We set the cut-off value for plasma D-dimer levels at 10 μg/ml. Although no recommended D-dimer target level is currently stated for anticoagulant therapy for cancer patients, this target value was supported by the findings of previous studies showing that the median lowest D-dimer level was 11 μg/ml in patients with at least one embolic signal detected by transcranial Doppler ultrasonography (21), and that most cancer patients with acute ischemic stroke who experienced recurrent stroke had a plasma D-dimer level ≥10 μg/ml (22). Cumulative rates of ischemic stroke recurrence and mortality at 30 days after admission in patients with and without post-treatment plasma D-dimer levels ≥10 μg/ml were compared using the Kaplan-Meier method and log-rank test. Logistic regression analysis was used to assess risk factors for poor outcomes and mortality at 30 days after admission. Backward stepwise elimination was used to select the independent variables. The diagnostic value of the D-dimer level for predicting poor outcomes was determined using receiver-operating characteristic (ROC) curve analysis. All statistical analyses were performed using JMP version 14.2.0 software (SAS Inc., Cary, NC, USA). Values of *P* < 0.05 were considered significant.

## Results

The clinical characteristics of study patients with cancer-associated stroke are shown in Table 1. The mean age was 73.7 years, and 125 patients (44.3%) were female. The most frequent type of cancer was lung cancer (27.0%). Adenocarcinoma was observed in 61.7% of patients, and approximately half of patients had systemic metastasis. Cancer was newly diagnosed after stroke in 7.4% of patients. Multiple infarcts on DWI were found in 68.4% of patients, and lesions involving multiple vascular territories were observed in 63.1% of patients. Median D-dimer plasma levels were 4.7 μg/ml before treatment and 3.1 μg/ml after treatment. Stroke treatment included heparin (51.4%), DOAC (7.8%), and warfarin (5.0%), whereas 35.8% of patients did not receive anticoagulants. Hemorrhagic events were observed in 21 patients (7.5%). Discontinuation of anticoagulants due to hemorrhagic events was observed in 2.8% of patients.

**Table 1:** Clinical characteristics of patients according to outcomes 30 days after admission.

		**Poor outcome (mRS score** **>3) at 30 days**		
**Characteristics**	**Overall (*n* = 282)**	**Yes (*n* = 135)**	**No (*n* = 147)**	***P***
Age, y	73.7 ± 10.9	75.3 ± 10.8	72.2 ± 10.8	0.02
Female	125 (44.3)	70 (51.2)	55 (37.4)	0.01
**Cancer type**				0.06
Lung	76 (27.0)	25 (18.5)	51 (34.7)	
Pancreatic	34 (12.1)	20 (14.8)	14 (9.5)	
Hepatobiliary	29 (10.3)	17 (12.6)	12 (8.2)	
Colorectal	24 (8.5)	10 (7.4)	14 (9.5)	
Gastric	20 (7.1)	9 (6.7)	11 (7.5)	
Gynecologic	8 (2.8)	5 (3.7)	3 (2.0)	
Others	91 (32.3)	49 (36.3)	42 (28.6)	
Adenocarcinoma	174 (61.7)	80 (59.3)	94 (64.0)	0.42
Systemic metastasis	143 (50.7)	79 (58.5)	64 (43.5)	0.01
Diagnosis of cancer after stroke	21 (7.4)	11 (8.2)	10 (6.8)	0.67
**Medical history**				
Hypertension	163 (57.8)	74 (54.8)	89 (60.5)	0.33
Diabetes mellitus	67 (23.8)	30 (22.2)	37 (25.2)	0.56
Dyslipidemia	95 (33.7)	39 (28.9)	56 (38.1)	0.10
Coronary artery disease	22 (7.8)	7 (5.2)	15 (10.2)	0.12
Prior stroke	43 (15.2)	24 (15.3)	19 (12.9)	0.26
Deep vein thrombosis	40 (14.2)	16 (11.9)	24 (16.3)	0.28
Current smoking	41 (14.5)	16 (11.9)	25 (17.0)	0.22
**Pre-admission treatment**				0.73
None	194 (68.8)	93 (68.9)	101 (68.7)	
Antiplatelet agents	34 (12.1)	18 (13.3)	16 (10.9)	
Anticoagulants	54 (19.1)	24 (17.8)	30 (20.4)	
Pre-admission mRS score	0 (0–2)	0 (0–2)	0 (0–1)	<0.001
NIHSS score on admission	7.8 ± 8.7	11.8 ± 9.5	4.1 ± 5.9	<0.001
**Multiple infarcts on DWI**	193 (68.4)	104 (77.0)	89 (60.5)	<0.01
Single vascular territory	15 (5.3)	5 (3.7)	10 (6.8)	
Multiple vascular territories	178 (63.1)	99 (73.3)	79 (53.7)	
Intravenous thrombolysis	7 (2.5)	2 (1.5)	5 (3.4)	0.30
**Stroke treatment**				<0.001
None	36 (12.8)	33 (24.4)	3 (2.0)	
Antiplatelet	65 (23.0)	19 (14.1)	46 (31.3)	
Warfarin	14 (5.0)	4 (3.0)	10 (6.8)	
DOAC	22 (7.8)	8 (5.9)	14 (9.5)	
Heparin	145 (51.4)	71 (52.6)	74 (50.3)	
Discontinuation of anticoagulants due to hemorrhagic events	5/181 (2.8)	4/83 (4.8)	1/98 (1.0)	0.12
Hemorrhagic events after admission	21 (7.5)	14 (10.4)	7 (4.8)	0.07
**Plasma D-dimer levels**				
Pre-treatment	4.7 (1.6–14.8)	9.43 (3.6–19.8)	2.5 (1.1–9.6)	<0.001
Post-treatment	3.1 (1.3–8.1)	6.3 (2.5–18.3)	1.9 (1.0–4.1)	<0.001

*DOAC, direct oral anticoagulant; DWI, diffusion-weighted imaging; mRS, modified Rankin Scale; NIHSS, National Institutes of Health Stroke Scale*.

*Values are numbers (%), means ± standard deviation, or medians (interquartile range)*.

Patients with poor outcomes 30 days after admission were significantly older (*P* = 0.02), with higher proportions of females (*P* = 0.01) and presence of systemic metastasis (*P* = 0.01). In addition, pre-admission mRS score (*P* < 0.001), NIHSS score on admission (*P* < 0.001), presence of multiple infarcts on DWI (*P* < 0.01), and D-dimer levels before (*P* < 0.001) and after treatment (*P* < 0.001) were significantly higher in patients with than in those without poor outcomes.

Clinical outcomes are shown in Table 2. At 30 days after admission, 135 patients (47.9%) showed poor outcomes. Recurrent ischemic stroke was identified in 28 patients (9.9%) within 30 days after admission. The cumulative mortality rate at 30 days was 12.4%. Of the 35 patients who died, the cause of death was cancer in 27 patients, ischemic stroke in four patients, pneumonia in two patients, cerebral hemorrhage in one patient, and pulmonary thromboembolism in one patient. Stroke treatment and ischemic stroke recurrence rates differed among participating hospitals, but no differences in the rates of poor outcomes or mortality were found (Supplementary Table 1).

**Table 2:** Clinical outcomes 30 days after admission according to the post-treatment plasma D-dimer level.

		**Post-treatment plasma D-dimer level** **≥10** **μg/ml**		
	**Overall (*n* = 282)**	**Yes (*n* = 55)**	**No (*n* = 227)**	***P***
Poor outcome (mRS score >3)	135 (47.9%)	45 (81.8%)	90 (39.7%)	<0.001
Cumulative recurrent ischemic stroke rate	28 (9.9%)	8 (14.6%)	20 (8.8%)	0.20
Cumulative mortality rate	35 (12.4%)	20 (36.4%)	15 (6.6%)	<0.001

*mRS, modified Rankin Scale*.

*Values are numbers (%)*.

**Figure 1:**
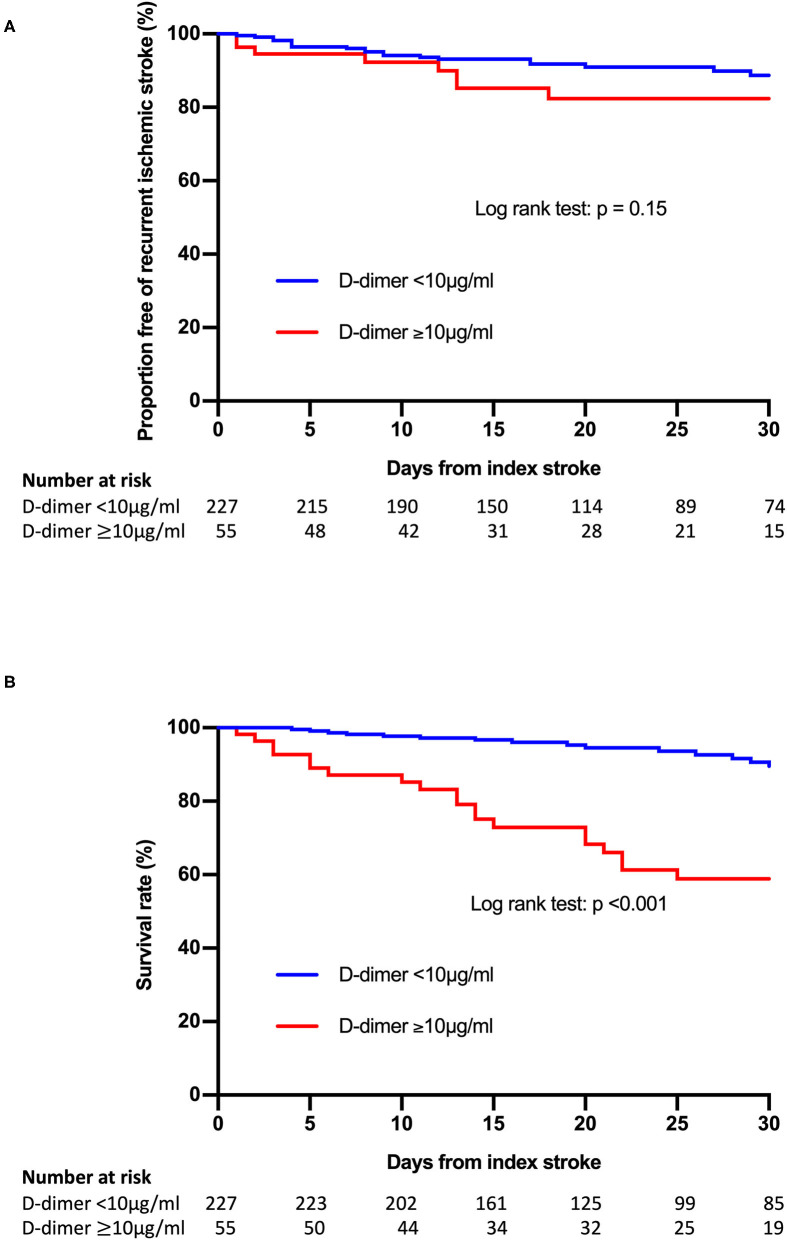
Kaplan-Meier curves of freedom from recurrence of ischemic stroke events **(A)** and survival **(B)** according to the post-treatment plasma D-dimer level.

Patients with post-treatment plasma D-dimer levels ≥10 μg/ml had significantly higher frequencies of poor outcomes (*P* < 0.001) and cumulative mortality rate 30 days after admission (*P* < 0.001) compared to those without. The rate of recurrent ischemic stroke within 30 days after admission tended to be higher in patients with post-treatment plasma D-dimer levels ≥10 μg/ml than in those without, but the difference was not significant. Kaplan-Meier analysis showed that a post-treatment plasma D-dimer level ≥10 μg/ml was significantly associated with poor survival 30 days after admission (log-rank test: *P* < 0.001) (Figure 1). There was no difference in pre-treatment D-dimer levels or clinical outcomes among the pre-admission treatment groups (Supplementary Table 2).

Multivariate analysis showed that a post-treatment plasma D-dimer level ≥10 μg/ml was independently associated with both poor outcomes (adjusted odds ratio [OR], 9.61; 95% confidence interval [CI], 3.60–25.70; *P* < 0.001) and mortality (adjusted OR, 9.38; 95% CI, 3.32–26.44; *P* < 0.001) (Table 3). Conversely, a pre-treatment plasma D-dimer level ≥10 μg/ml was not associated with these outcomes. Other variables including age (adjusted OR, 1.04; 95% CI, 1.01–1.07; *P* < 0.05), pre-admission mRS score (adjusted OR, 1.30; 95% CI, 1.01–1.68; *P* < 0.05), NIHSS score on admission (adjusted OR, 1.16; 95% CI, 1.11–1.22; *P* < 0.001), and presence of multiple infarcts on DWI (adjusted OR, 2.81; 95% CI, 1.38–5.73; *P* < 0.01) were associated with poor outcomes, and the presence of multiple infarcts on DWI (adjusted OR, 3.69; 95% CI, 1.06–12.79; *P* < 0.05) was associated with mortality.

**Table 3:** Univariate and multivariate regression analyses for poor outcomes and mortality 30 days after admission.

	**Poor outcome (mRS score** **>3)**		**Mortality**	
	**Univariate OR (95% CI)**	**Multivariate OR (95% CI)**	**Univariate OR (95% CI)**	**Multivariate OR (95% CI)**
Age	1.03 (1.00–1.05)^*^	1.04 (1.01–1.07)^*^	1.00 (0.97–1.03)	1.00 (0.96–1.04)
Female	0.56 (0.35–0.89)^*^	0.69 (0.36–1.32)	0.72 (0.35–1.47)	0.64 (0.27–1.55)
Systemic metastasis	1.83 (1.14–2.94)^*^	1.75 (0.89–3.44)	3.21 (1.45–7.13)^**^	1.77 (0.66–4.74)
Pre-admission mRS score	1.50 (1.23–1.83)^***^	1.30 (1.01–1.68)^*^	1.28 (1.01–1.63)^*^	1.26 (0.95–1.67)
NIHSS score on admission (per 1-point increase)	1.15 (1.10–1.20)^***^	1.16 (1.11–1.22)^***^	1.04 (1.00–1.08)^*^	1.04 (0.99–1.09)
Multiple infarcts on DWI	2.19 (1.30–3.68)^**^	2.81 (1.38–5.73)^**^	4.07 (1.39–11.9)^**^	3.69 (1.06–12.79)^*^
Pre-treatment D-dimer level ≥10 μg/ml	2.82 (1.69–4.71)^***^	1.08 (0.51–2.27)	3.96 (1.89–8.27)^***^	1.05 (0.36–3.08)
Post-treatment D-dimer level ≥10 μg/ml	6.85 (3.28–14.3)^***^	9.61 (3.60–25.70)^***^	8.08 (3.78–17.3)^***^	9.38 (3.32–26.44)^***^

*CI, confidence interval; DWI, diffusion-weighted imaging; mRS, modified Rankin Scale; NIHSS, National Institutes of Health Stroke Scale; OR, odds ratio*.

* *P < 0.05*;

** *P < 0.01*;

*** *P < 0.001*.

The ROC curve analyses showed that the pre- and post-treatment D-dimer levels to predict poor outcomes were 2.60 μg/ml (area under the curve [AUC] = 0.701, *P* < 0.001) and 2.83 μg/ml (AUC = 0.743, *P* < 0.001), respectively. Pre- and post-D-dimer levels to predict mortality were 4.40 μg/ml (AUC = 0.75, *P* < 0.01) and 3.40 μg/ml (AUC = 0.80, *P* < 0.01), respectively. Multivariate analysis showed the same tendency when the cutoff value of D-dimer was set to ≥3 μg/ml (Supplementary Table 3).

**Figure 2:**
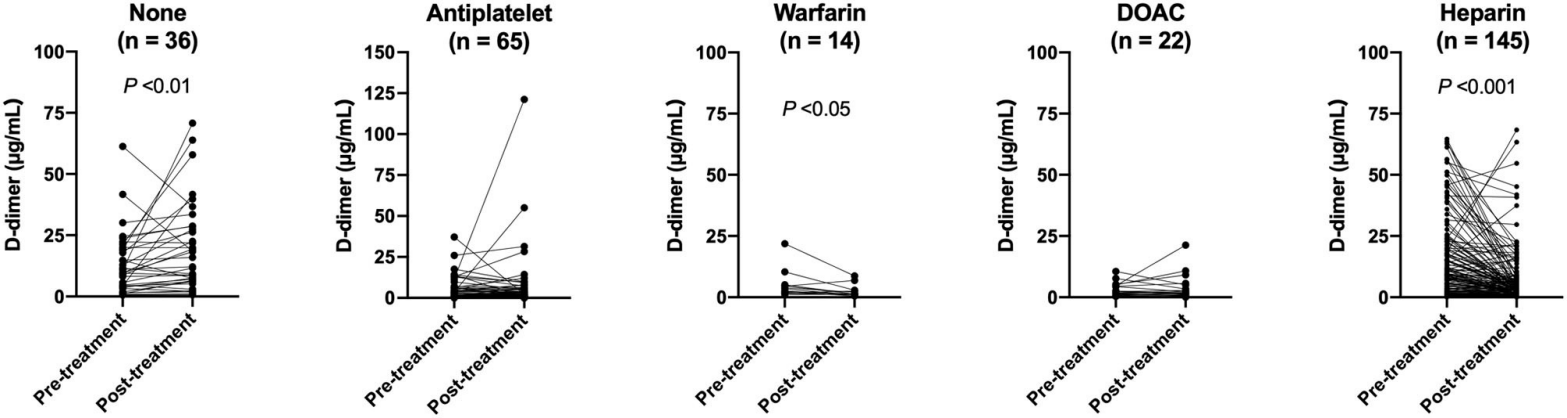
Changes in plasma D-dimer levels from before to after treatment.

Changes in the plasma D-dimer level from pre-treatment to post-treatment were compared among stroke treatment groups. Patients who received heparin had higher pre-treatment plasma D-dimer levels compared with those who were treated with other anticoagulants such as warfarin or DOACs (Table 4). Plasma D-dimer levels were significantly increased in patients with no treatment (*P* < 0.01), and they were decreased in those who received warfarin (*P* < 0.05) or heparin (*P* < 0.001) (Figure 2).

**Table 4:** Pre- and post-treatment plasma D-dimer levels and changes in plasma D-dimer levels in each stroke treatment group.

	**None**	**Antiplatelet**	**Warfarin**	**DOAC**	**Heparin**	***P***
	**(*n* = 36)**	**(*n* = 65)**	**(*n* = 14)**	**(*n* = 22)**	**(*n* = 145)**	
**Plasma D-dimer (μg/ml)**						
Pre-treatment	9.6 (4.0–19.2)	1.9 (1.3–5.3)	3.0 (1.8–4.7)	2.1 (1.1–4.5)	9.1 (2.6–21.3)	<0.001
Post-treatment	14.0 (6.3–28.3)	2.3 (1.1–5.3)	1.3 (1.0–2.6)	1.5 (0.7–3.8)	3.2 (1.4–7.5)	<0.01
Change (post – pre)	6.5 ± 16.3	2.0 ± 16.1	−2.5 ± 3.9	0.4 ± 4.3	−7.7 ± 15.4	<0.001

*DOAC, direct oral anticoagulant*.

*Values are means ± standard deviation or medians (interquartile range)*.

Clinical characteristics of patients in each antithrombotic treatment group are shown in Supplementary Table 4. The frequency of systemic metastasis was highest in the heparin group, followed by the no antithrombotic group. The NIHSS score on admission was higher in the no antithrombotic and DOAC groups compared with the other groups. Table 5 shows rates of hemorrhagic events and clinical outcomes in each antithrombotic treatment group. Hemorrhagic events were the most frequent, and clinical outcomes were the worst in the no antithrombotic group. In patients who received antithrombotic therapy, the heparin group showed fewer hemorrhagic events (4.1%) compared with the antiplatelet (4.6%), warfarin (7.1%), and DOAC (13.6%) groups. Clinical outcomes, however, were worse in the heparin group compared with these other groups.

**Table 5:** Rates of hemorrhagic events within 30 days after admission and clinical outcomes in each antithrombotic treatment group.

	**Antithrombotic treatment**					
	**None**	**Antiplatelet**	**Warfarin**	**DOAC**	**Heparin**	***P***
	**(*n* = 36)**	**(*n* = 65)**	**(*n* = 14)**	**(*n* = 22)**	**(*n* = 145)**	
**Hemorrhagic event**	8 (22.2)	3 (4.6)	1 (7.1)	3 (13.6)	6 (4.1)	<0.01
Cerebral hemorrhage	1 (2.8)	1 (1.5)	0 (0)	1 (4.6)	0 (0)	
Gastrointestinal bleeding	1 (2.8)	1 (1.5)	1 (7.1)	1 (4.6)	4 (2.8)	
Urinary tract bleeding	1 (2.8)	1 (1.5)	0 (0)	1 (4.6)	1 (0.7)	
Intraperitoneal bleeding	2 (5.6)	0 (0)	0 (0)	0 (0)	0 (0)	
Intratumor bleeding	3 (8.3)	0 (0)	0 (0)	0 (0)	1 (0.7)	
**Clinical outcome at 30 days**						
Poor outcome (mRS score >3)	33 (91.7)	19 (29.2)	4 (28.6)	8 (36.4)	71 (49.0)	<0.001
Cumulative recurrent ischemic stroke rate	8 (22.2)	4 (6.2)	1 (7.1)	1 (4.6)	14 (9.7)	0.09
Cumulative mortality rate	9 (25.0)	1 (1.5)	1 (7.1)	1 (4.6)	23 (15.9)	<0.01

*DOAC, direct oral anticoagulant*.

*Values are numbers (%)*.

## Discussion

The present study showed that a high plasma D-dimer level post-treatment was associated with poor outcomes and a higher mortality rate 30 days after admission in patients with cancer-associated stroke independent of other covariates including age, systemic metastasis, pre-admission mRS score, NIHSS score on admission, multiple infarcts on DWI, and pre-treatment D-dimer levels. Anticoagulant therapy with heparin had a significant effect on reducing plasma D-dimer levels in patients with high D-dimer levels. The present results suggest the importance of correcting hypercoagulability with anticoagulation therapy to achieve better short-term outcomes for patients with cancer-associated stroke.

Patients with cancer have poor outcomes after acute ischemic stroke (8, 13, 16, 23, 26, 27). In the present study, approximately half of the patients had poor outcomes. Importantly, the rate of poor outcomes differed significantly between patients with post-treatment plasma D-dimer levels ≥10 μg/ml (81.8%) and those without (39.7%, *P* < 0.001). Multivariate analysis showed that a post-treatment, but not pre-treatment, plasma D-dimer level ≥10 μg/ml was independently associated with poor outcomes.

In-hospital mortality rates are reportedly higher in ischemic stroke patients with active cancer than in those without (8, 16, 26). A recent large, observational, Korean study reported a mortality rate of 18.3% at 1 month and 71.6% at 1 year in patients with active cancer (27). In line with the previous study, the mortality rate at 30 days was 12.4% in the present study. In addition, it was found that the 30-day mortality rate was significantly higher in patients with a post-treatment plasma D-dimer level ≥10 μg/ml (36.4%) than in those without (6.6%, *P* < 0.001). Although a pre-treatment plasma D-dimer level ≥10 μg/ml was associated with 30-day mortality on univariate analysis, this association disappeared after adjusting for other covariates, including post-treatment D-dimer levels. In contrast, multivariate analysis showed that a post-treatment plasma D-dimer level ≥10 μg/ml was independently associated with the 30-day mortality rate. These findings were in line with results from a previous study in which post-treatment, but not pre-treatment, D-dimer levels were independently associated with poor 1-year survival (23).

In patients with acute ischemic stroke and cancer, cumulative rates of recurrent ischemic stroke have been reported as 7, 13, and 16% at 1, 3, and 6 months, respectively (27). Similarly, the cumulative recurrent ischemic stroke rate at 30 days was 9.9% in the present study. Although this rate was higher in patients with a post-treatment plasma D-dimer level ≥10 μg/ml than in those without, the difference between these groups was not significant. These results suggest that recurrence of ischemic stroke is not necessarily the cause of poor prognosis or death. In fact, most causes of death were due to cancer in the present study. Hypercoagulation may adversely affect the prognosis of cancer patients through multi-organ thrombosis.

Among cancer types, hepatobiliary and pancreatic cancers have been associated with higher overall and 1-year mortality rates in patients with ischemic stroke (23). Additionally, melanoma and lung/respiratory tract cancers have been identified as the strongest predictors of death in young stroke patients with cancer during a median follow-up of 10 years (10). A population-based cohort study showed that lung cancer was associated with an increased risk of subsequent stroke within 1 year after diagnosis for men and 2 years after diagnosis for women (1). In contrast to these previous studies, the cancer type was not associated with poor outcomes or mortality 30 days after admission in the present patients. Similarly, no association was found between cancer type and early neurological deterioration in patients with acute ischemic stroke and cancer (28). Thus, cancer type may be related to long-term outcomes or risk of stroke in patients with cancer. Regarding the underlying histology, adenocarcinoma was found in 61.7% of the present patients, similar to the results of previous studies (8, 23, 27). Adenocarcinoma has been independently associated with the presence of embolic signals on transcranial Doppler ultrasonography (21) and recurrent thromboembolism including ischemic stroke (27) in patients with cancer and acute ischemic stroke. However, the coagulation status rather than cancer type or histology was related to outcomes in the present patients.

Systemic metastasis was found in approximately half of patients in the present study, again similar to previous studies (13, 21, 23, 27). In the present study, systemic metastasis was independently associated with poor outcomes. Systemic metastasis is associated with overall and 1-year mortality in patients with ischemic stroke (23). Advanced stage is associated with increased short-term risk of arterial thromboembolism including ischemic stroke in patients with newly diagnosed cancer in population-based samples (4). Such findings suggest that an advanced cancer stage affects the risk and outcomes of ischemic stroke. Moreover, the presence of multiple infarcts on DWI was independently associated with poor outcomes and mortality in the present study. The high prevalence of lesions involving multiple vascular territories found in the present study has been reported as a characteristic finding of cancer-related ischemic stroke (8, 14, 16–18, 29).

Anticoagulation treatment reduces plasma D-dimer levels in patients with cancer (21, 23). However, the effects of different types of anticoagulants on D-dimer levels have not been clarified in these studies. Pre- and post-treatment D-dimer plasma levels were assessed in each anticoagulant group in the present study, and pre-treatment D-dimer levels were the highest in patients who received heparin. D-dimer levels decreased from before to after treatment in patients who received heparin or warfarin, and the value was greater in patients who received heparin than in those who received warfarin. D-dimer levels were not significantly changed in patients who received DOAC, and they were significantly increased in those who received no stroke treatment. However, pre-treatment D-dimer levels were different among the groups, and it was not possible to determine which anticoagulant was more effective for reducing D-dimer levels. Nonetheless, the present findings suggest that heparin has significant effects to improve the hypercoagulable state in patients with cancer-associated stroke. A retrospective, observational study reported that low-molecular-weight heparin (LMWH) was more effective than warfarin for lowering plasma D-dimer levels in patients with cancer-associated stroke (22). Another retrospective study showed that clinical outcomes, including early neurologic deterioration, recurrent ischemic stroke, 3-month mRS score, and 90-day mortality, did not differ between patients treated with DOAC and those treated with LMWH in a cohort with cryptogenic ischemic stroke and active cancer (30). However, the number of patients included in these studies was small, and further prospective studies with larger samples are needed to confirm the results.

We previously reported that medical conditions, including hemorrhagic complications, hinder heparin treatment in patients with cancer-associated stroke (31). Patients with cancer have an increased risk of hemorrhagic complications due to cancer-related factors including local injury due to tumor invasion, thrombocytopenia induced by chemotherapy, and surgery (32). Although hemorrhagic complications are the major concern in patients receiving antithrombotic therapy, the frequency of hemorrhagic events in patients with cancer-associated stroke has been reported in only a few studies with small sample sizes (22, 30) (Supplementary Table 5). In the present study, the overall rate of hemorrhagic events was 7.5% within 30 days after admission. The heparin group showed the fewest hemorrhagic events (4.1%) among patients receiving antithrombotic therapy. Notably, hemorrhagic events were more common in patients without antithrombotic therapy (22.2%) than in those receiving antithrombotic therapy. The no antithrombotic treatment group showed the second highest rate of systemic metastasis after the heparin group, and it had the highest stroke severity on admission of the groups. Thus, antithrombotic drugs may not have been administered to patients at high risk of bleeding in the present study. Currently, no clear standards have been set by which to determine whether patients should be given antithrombotic drugs following cancer-associated stroke. Further research is needed to determine criteria for the indication of anticoagulation therapy. Despite the significant effect of heparin treatment on lowering D-dimer levels, the clinical outcomes of the heparin group were worse than of the other antithrombotic groups. This might be due to background factors such as a high proportion of patients with systemic metastases in the heparin group.

The strengths of the present study included the multicenter design and the large number of patients. However, the present study has some limitations. First, it was a retrospective, observational study, and causality cannot be inferred from the data. Additionally, those patients for whom D-dimer levels were not measured after stroke treatment were excluded, which caused some degree of selection bias. Second, although heparin improved hypercoagulability in patients with cancer-associated stroke, one cannot draw any conclusions about the appropriate dose of heparin. The present findings suggest the importance of adjusting the heparin dose to reach the target D-dimer level. However, the analysis focused on short-term outcomes. It is often difficult to continue heparin treatment for an extended time. The effects of heparin on long-term outcomes need to be investigated in further research. Third, because LMWH is not approved for cancer-associated thrombosis in Japan, either continuous intravenous infusion of unfractionated heparin or twice-daily subcutaneous injection of heparin calcium was used in the present study. Fourth, the present study lacked detailed data on cancer stages. However, the analysis included systemic metastases (stage 4), which have been shown to be associated with prognosis in many previous studies. Finally, cancer treatment after stroke was not included in the analysis. However, cancer treatment may affect long-term prognosis rather than short-term prognosis, as evaluated in the present study.

In conclusion, a high D-dimer level after stroke treatment was associated with poor short-term outcomes in patients with cancer-associated stroke. Heparin may effectively reduce D-dimer levels in patients in a hypercoagulable state. Further prospective studies are needed to explore the optimal D-dimer levels to improve the prognosis of patients with cancer-associated stroke.

## Data Availability Statement

The original contributions presented in the study are included in the article/Supplementary Material, further inquiries can be directed to the corresponding author/s.

## Ethics Statement

The studies involving human participants were reviewed and approved by Juntendo University Ethics Review Committee. Written informed consent for participation was not required for this study in accordance with the national legislation and the institutional requirements.

## Author Contributions

KY and NH: designed study. SN, HK, KY, RT, TK, NK, KH, NM, YU, and MW: data acquisition. SN, HK, and KY: analyzed data and drafted manuscript. KY, RT, TK, NK, KH, NM, YU, MW, TH, SF, TU, and NH: reviewed and edited manuscript. All authors contributed to the article and approved the submitted version.

## Funding

This study was supported by institutional funds.

## Conflict of Interest

The authors declare that the research was conducted in the absence of any commercial or financial relationships that could be construed as a potential conflict of interest.

## Publisher's Note

All claims expressed in this article are solely those of the authors and do not necessarily represent those of their affiliated organizations, or those of the publisher, the editors and the reviewers. Any product that may be evaluated in this article, or claim that may be made by its manufacturer, is not guaranteed or endorsed by the publisher.
