# Supplementary material for: Post-Treatment Plasma D-Dimer Levels Are Associated With Short-Term Outcomes in Patients With Cancer-Associated Stroke
Source: Front Neurol. 2022 Apr 4;13:868137. doi: 10.3389/fneur.2022.868137 (PMC9015657; doi:10.3389/fneur.2022.868137)
Supplement: Supplementary file 1 [file Table_1.DOCX]

Supplementary Material

# Supplementary Table 1. Stroke treatment and clinical outcomes 30 days after admission in each hospital

|  | Juntendo University Hospital | Juntendo University Urayasu Hospital | Kyorin University Hospital | Jichi Medical University Hospital | Shin-Oyama City Hospital | *P* |
| --- | --- | --- | --- | --- | --- | --- |
|  | (n = 126) | (n = 27) | (n = 88) | (n = 30) | (n = 11) |  |
| **Stroke treatment** |  |  |  |  |  | <0.001 |
| None | 16 (12.7) | 3 (11.1) | 14 (15.9) | 1 (3.3) | 2 (18.2) |  |
| Antiplatelet | 38 (30.2) | 12 (44.4) | 11 (12.5) | 4 (13.3) | 0 (0) |  |
| Warfarin | 12 (9.5) | 2 (7.4) | 0 (0) | 0 (0) | 0 (0) |  |
| DOAC | 9 (7.1) | 5 (18.5) | 7 (8.0) | 0 (0) | 1 (9.1) |  |
| Heparin | 51 (40.5) | 5 (18.5) | 56 (63.6) | 25 (83.3) | 8 (72.7) |  |
| **Outcome 30 days after admission** |  |  |  |  |  |  |
| Poor outcome (mRS score >3) | 55 (43.7) | 11 (40.7) | 46 (52.3) | 14 (46.7) | 9 (81.8) | 0.12 |
| Cumulative recurrent ischemic stroke rate | 9 (7.1) | 1 (3.7) | 17 (19.3) | 1 (3.3) | 0 (0) | <0.01 |
| Cumulative mortality | 16 (12.7) | 2 (7.4) | 13 (14.8) | 1 (3.3) | 3 (27.3) | 0.23 |

DOAC, direct oral anticoagulant; mRS, modified Rankin Scale
