# Supplementary material for: Post-Treatment Plasma D-Dimer Levels Are Associated With Short-Term Outcomes in Patients With Cancer-Associated Stroke
Source: Front Neurol. 2022 Apr 4;13:868137. doi: 10.3389/fneur.2022.868137 (PMC9015657; doi:10.3389/fneur.2022.868137)
Supplement: Supplementary file 2 [file Table_2.DOCX]

Supplementary Material

# Supplementary Table 2. Pre-treatment plasma D-dimer levels and clinical outcomes 30 days after admission in each pre-admission treatment group

|  | None | Antiplatelet agents | Anticoagulants | *P* |
| --- | --- | --- | --- | --- |
|  | (n = 194) | (n = 34) | (n = 54) |  |
| Pre-treatment plasma D-dimer level | 4.6 (1.5 – 15.3) | 5.6 (2.3 – 14.3) | 5.0 (1.9 – 11.5) | 0.71 |
| Clinical outcome at 30 days |  |  |  |  |
| Poor outcome (mRS score >3) | 93 (47.9) | 18 (52.9) | 24 (44.4) | 0.74 |
| Cumulative recurrent ischemic stroke rate | 18 (9.3) | 3 (8.8) | 7 (13.0) | 0.71 |
| Cumulative mortality rate | 23 (11.9) | 2 (5.9) | 10 (18.5) | 0.20 |

mRS, modified Rankin Scale.
