# Supplementary material for: Post-Treatment Plasma D-Dimer Levels Are Associated With Short-Term Outcomes in Patients With Cancer-Associated Stroke
Source: Front Neurol. 2022 Apr 4;13:868137. doi: 10.3389/fneur.2022.868137 (PMC9015657; doi:10.3389/fneur.2022.868137)
Supplement: Supplementary file 3 [file Table_3.DOCX]

Supplementary Material

# Supplementary Table 3. Univariate and multivariate regression analyses for poor outcomes and mortality 30 days after admission

|  | Poor outcome (mRS score >3) | | Mortality | |
| --- | --- | --- | --- | --- |
|  | Univariate OR  (95% CI) | Multivariate OR  (95% CI) | Univariate OR  (95% CI) | Multivariate OR  (95% CI) |
| Age | 1.03 (1.00–1.05)* | 1.04 (1.01–1.07)* | 1.00 (0.97–1.04) | 1.00 (0.97–1.04) |
| Female | 0.56 (0.35–0.89)* | 0.79 (0.41–1.54) | 0.72 (0.36–1.47) | 0.76 (0.32–1.78) |
| Systemic metastasis | 1.83 (1.14–2.94)* | 1.73 (0.88–3.40) | 3.21 (1.45–7.13)** | 1.86 (0.74–4.71) |
| Pre-admission mRS score | 1.50 (1.23–1.83)*** | 1.42 (1.09–1.87)** | 1.28 (1.01–1.63) | 1.26 (0.95–1.67) |
| NIHSS score on admission (per 1-point increase) | 1.15 (1.10–1.20)*** | 1.17 (1.11–1.23)*** | 1.04 (1.00–1.08)* | 1.04 (0.99–1.09) |
| Multiple infarcts on DWI | 2.19 (1.30–3.68)** | 1.97 (0.95–4.10) | 4.07 (1.39–11.90)* | 2.66 (0.82–8.59) |
| Pre-treatment D-dimer level ≥3 μg/ml | 4.09 (2.41–6.93)*** | 2.01 (0.88–4.60) | 24.32 (3.28–180.53)** | 6.30 (0.75–53.16) |
| Post-treatment D-dimer level ≥3 μg/ml | 4.32 (2.62–7.11)*** | 4.73 (2.20–10.17)*** | 9.04 (3.10–26.38)*** | 6.19 (1.65–23.22)** |

CI, confidence interval; DWI, diffusion-weighted imaging; mRS, modified Rankin Scale; NIHSS, National Institutes of Health Stroke Scale; OR, odds ratio.

**P* < 0.05; ***P* < 0.01; ****P* < 0.001.
