# Supplementary material for: Post-Treatment Plasma D-Dimer Levels Are Associated With Short-Term Outcomes in Patients With Cancer-Associated Stroke
Source: Front Neurol. 2022 Apr 4;13:868137. doi: 10.3389/fneur.2022.868137 (PMC9015657; doi:10.3389/fneur.2022.868137)
Supplement: Supplementary file 4 [file Table_4.DOCX]

Supplementary Material

# Supplementary Table 4. Clinical characteristics of patients in each antithrombotic treatment group

|  | Antithrombotic treatment | | | | |  |
| --- | --- | --- | --- | --- | --- | --- |
|  | None | Antiplatelet | Warfarin | DOAC | Heparin | *P* |
|  | (n = 36) | (n = 65) | (n = 14) | (n = 22) | (n = 145) |  |
| Age, y | 74.2 ± 12.0 | 74.0 ± 10.4 | 66.9 ± 10.4 | 78.1 ± 8.5 | 73.4 ± 10.9 | 0.05 |
| Female | 20 (55.6) | 16 (24.6) | 8 (57.1) | 11 (55.0) | 70 (48.3) | <0.01 |
| **Cancer type** |  |  |  |  |  | 0.06 |
| Lung | 7 (19.4) | 17 (26.2) | 3 (21.4) | 6 (27.3) | 43 (29.7) |  |
| Pancreatic | 5 (13.9) | 4 (6.2) | 0 (0) | 0 (0) | 25 (17.2) |  |
| Hepatobiliary | 2 (5.6) | 7 (10.8) | 1 (7.1) | 0 (0) | 19 (13.1) |  |
| Colorectal | 1 (2.8) | 9 (13.9) | 3 (21.4) | 2 (9.1) | 9 (6.2) |  |
| Gastric | 3 (8.3) | 5 (7.7) | 1 (7.1) | 1 (4.6) | 10 (6.9) |  |
| Gynecological | 1 (2.8) | 0 (0) | 1 (7.1) | 1 (4.6) | 5 (3.5) |  |
| Others | 17 (47.2) | 23 (35.4) | 5 (35.7) | 12 (54.6) | 34 (23.5) |  |
| Adenocarcinoma | 17 (47.2) | 35 (53.9) | 8 (57.1) | 10 (45.5) | 104 (71.7) | <0.01 |
| Systemic metastasis | 20 (55.6) | 18 (27.7) | 1 (7.1) | 5 (22.7) | 99 (68.3) | <0.001 |
| Diagnosis of cancer after stroke | 1 (2.8) | 1 (1.5) | 1 (7.1) | 3 (13.6) | 15 (10.3) | 0.12 |
| **Medical history** |  |  |  |  |  |  |
| Hypertension | 17 (47.2) | 35 (53.9) | 7 (50.0) | 16 (72.7) | 88 (60.7) | 0.30 |
| Diabetes | 5 (13.9) | 19 (23.2) | 2 (14.3) | 4 (18.2) | 37 (25.5) | 0.36 |
| Dyslipidemia | 8 (22.2) | 26 (40.0) | 3 (21.4) | 11 (50.0) | 95 (33.7) | 0.14 |
| Coronary artery disease | 1 (2.8) | 7 (10.8) | 1 (7.1) | 2 (9.1) | 11 (7.6) | 0.71 |
| Prior stroke | 4 (11.1) | 8 (12.3) | 5 (35.7) | 6 (27.3) | 20 (13.8) | 0.09 |
| Deep vein thrombosis | 2 (5.6) | 2 (3.1) | 3 (21.4) | 4 (18.2) | 29 (20.0) | <0.01 |
| Current smoking | 3 (8.3) | 14 (21.5) | 4 (28.6) | 2 (9.1) | 18 (12.4) | 0.14 |
| **Pre-admission treatment** |  |  |  |  |  |  |
| None | 27 (75.0) | 49 (75.4) | 10 (71.4) | 13 (59.1) | 95 (65.5) | <0.01 |
| Antiplatelet agents | 7 (19.4) | 11 (16.9) | 0 (0) | 4 (18.2) | 12 (8.3) |  |
| Anticoagulants | 2 (5.6) | 5 (7.7) | 4 (28.6) | 5 (22.7) | 38 (26.2) |  |
| NIHSS score on admission | 10.8 ± 9.9 | 5.9 ± 8.5 | 6.0 ± 5.2 | 10.8 ± 9.3 | 7.6 ± 8.5 | 0.04 |
| Multiple infarcts on DWI | 26 (72.2) | 31 (47.7) | 6 (42.9) | 9 (40.9) | 121 (83.5) | <0.001 |
| Single vascular territory | 1 (2.8) | 7 (10.8) | 2 (14.3) | 1 (4.5) | 4 (2.8) |  |
| Multiple vascular territories | 25 (69.4) | 24 (36.9) | 4 (28.6) | 8 (36.4) | 117 (80.7) |  |

DOAC, direct oral anticoagulant; DWI, diffusion-weighted imaging; NIHSS, National Institutes of Health Stroke Scale
