# Supplementary material for: Post-Treatment Plasma D-Dimer Levels Are Associated With Short-Term Outcomes in Patients With Cancer-Associated Stroke
Source: Front Neurol. 2022 Apr 4;13:868137. doi: 10.3389/fneur.2022.868137 (PMC9015657; doi:10.3389/fneur.2022.868137)
Supplement: Supplementary file 5 [file Table_5.DOCX]

Supplementary Material

# Supplementary Table 5. Incidence of hemorrhagic events in patients with cancer-associated stroke treated with anticoagulants in earlier studies

| Authors | Follow-up period | Hemorrhagic events | |
| --- | --- | --- | --- |
| Jang et al. [22] | 4.9 months (mean) | LMWH (n = 29) | Warfarin (n = 50) |
|  |  | 6.9% | 10.0% |
| Nam et al. [30] | 90 days | LMWH (n = 41) | DOAC (n = 7) |
|  |  | 39% | 29% |

DOAC, direct oral anticoagulant; LMWH, low-molecular-weight heparin
